# Anti-Cancer Effect of 3-Hydroxy-β-Ionone Identified from *Moringa oleifera* Lam. Leaf on Human Squamous Cell Carcinoma 15 Cell Line

**DOI:** 10.3390/molecules25163563

**Published:** 2020-08-05

**Authors:** Thitiya Luetragoon, Rungnapa Pankla Sranujit, Chanai Noysang, Yordhathai Thongsri, Pachuen Potup, Nungruthai Suphrom, Nitra Nuengchamnong, Kanchana Usuwanthim

**Affiliations:** 1 Cellular and Molecular Immunology Research Unit, Faculty of Allied Health Sciences, Naresuan University, Phitsanulok 65000, Thailand; nok.hong.yok49@gmail.com (T.L.); yordhathai.k@gmail.com (Y.T.); pachuenp@nu.ac.th (P.P.); 2 Faculty of Medicine, School of Human Development and Health, University of Southampton, Southampton SO16 6YD, UK; 3 Thai Traditional Medicine College, Rajamangala University of Technology Thanyaburi, Pathum Thani 12130, Thailand; rungnapa_s@rmutt.ac.th (R.P.S.); chanai_n@rmutt.ac.th (C.N.); 4 Department of Chemistry, Faculty of Science and Center of Excellence for Innovation in Chemistry, Naresuan University, Phitsanulok 65000, Thailand; suphrom.n1@gmail.com; 5 Science Laboratory Centre, Faculty of Science, Naresuan University, Phitsanulok 65000, Thailand; Nitran@nu.ac.th

**Keywords:** squamous cell carcinoma, *Moringa oleifera*, anti-cancer, 3-hydroxy-β-ionone

## Abstract

Squamous cell carcinoma is the most common type of head and neck cancer worldwide. Radiation and chemotherapy are general treatments for patients; however, these remedies can have adverse side effects and tumours develop drug resistance. Effective treatments still require improvement for cancer patients. Here, we investigated the anti-cancer effect of *Moringa oleifera* (MO) Lam. leaf extracts and their fractions, 3-hydroxy-β-ionone on SCC15 cell line. SCC15 were treated with and without MO leaf extracts and their fractions. MTT assay was used to determine cell viability on SCC15. Cell cycle and apoptosis were evaluated by the Muse™ Cell Analyser. Colony formation and wound closure analysis of SCC15 were performed in 6-well plates. Apoptosis markers were evaluated by immunoblotting. We found that Moringa extracts and 3-HBI significantly inhibited proliferation of SCC15. Moreover, they induced apoptosis and cell cycle arrest at G2/M phase in SCC15 compared to the untreated control. MO extracts and 3-HBI also inhibited colony formation and cell migration of SCC15. Furthermore, we observed the upregulation of cleaved caspase-3 and Bax with downregulation of anti-apoptotic Bcl-2, indicating the induction of cancer cell apoptosis. Our results revealed that MO extracts and 3-HBI provided anti-cancer properties by inhibiting progression and inducing apoptosis of SCC15.

## 1. Introduction

Cancer is a noncommunicable disease and the leading cause of death worldwide. Around 18.1 million new cases of cancer and 9.6 million deaths from the disease were reported in 2018 [1]. Global incidences of head and neck squamous cell carcinoma (HNSCC) were reported at more than 830,000 cases with 430,000 deaths each year [2]. Major risk factors of HNSCC are high smoking levels and alcohol consumption [3]. HNSCC arises from the mucosal surfaces at various sites including skin, nasal cavity, paranasal sinuses, oral cavity, salivary glands, pharynx and larynx [4]. Treatment for HNSCC usually involves therapy with surgery, radiation, chemotherapy, targeted therapy, immunotherapy and combination therapy [2]. Nevertheless, these treatments result in adverse effects including nausea, vomiting, fatigue, mucositis, dysphagia and dermatitis [5]. Major hallmarks during cancer development include the ability to proliferate, evade apoptosis, uncontrolled replicative potential, induction of angiogenesis and tissue invasion and metastasis to other organs. Hence, many drugs and treatment methods have been developed to interfere with each step and impede tumour growth and progression [6]. The goal of medical scientists and researchers is to improve the best treatment for good quality of life for cancer patients. Therefore, targeted therapy and alternative low toxic treatments have received intense focus. Several studies have examined the effects of natural products against tumours by inducing apoptosis via the P53 tumour suppressor and reactive oxygen species production [7,8,9].

**Figure 1:**
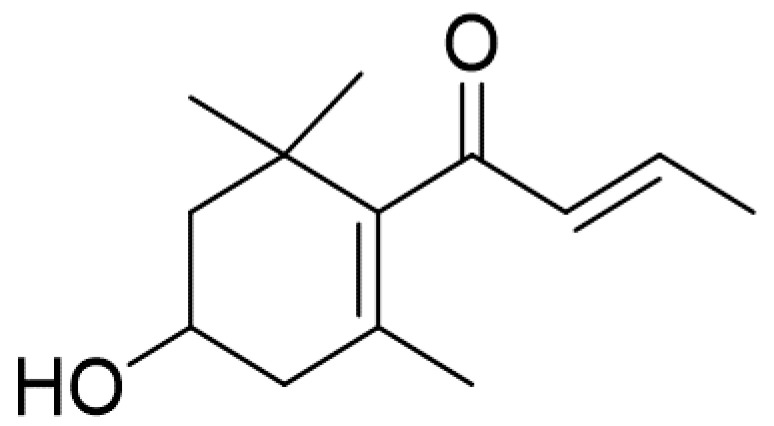
Structure of 3-hydroxy-β-ionone.

*Moringa oleifera* Lam. (MO) is known as the miracle tree and is widely cultivated in Asia and Africa. All the different parts of MO have been reported to have medicinal use [10]. The leaves of this plant have been intensively studied and contain high amounts of vitamins, carotenoids, polyphenols, phenolic acids, flavonoids, alkaloids, glucosinolates, isothiocyanates, tannins and saponins [11]. Many studies have reported on the biological activities of Moringa leaf such as antidiabetic [12], antioxidant [13], antibacterial [14] and kidney and hepatic protective effect [15,16]. Our previous study demonstrated that MO leaf extract provides anti-inflammatory potential by reducing the production of pro-inflammatory mediators such as interleukin-6, tumour necrosis factor-α and cyclooxygenase-2 via inactivation of NF-κB, inhibiting both IκB-α degradation and nuclear translocation of p65 [17,18]. 3-hydroxy-β-ionone (3-HBI) derived from MO leaf extract (Figure 1) had potent anti-inflammatory effects [17], while *in vitro* studies showed that soluble extract from MO leaf induced apoptosis and inhibited tumour cell growth in human non-small cell lung cancer A549 and human hepatocellular carcinoma HepG2 cells [19,20]. Another *in vitro* study reported that MO leaf extract and its compounds including eugenol, isopropyl isothiocyanate, D-allose and hexadeconoic acid ethyl ester decreased cell motility and colony formation, inhibited cell growth and triggered cell apoptosis against breast cancer and colorectal cancer cell lines [21]. Astragalin and isoquercetin from bioactive fractions of *M. oleifera* leaf extract suppressed proliferation of HCT116 colon cancer cells by downregulation of ERK1/2 phosphorylation [22]. In addition, glucomoringin from *Moringa oleifera* induced oxidative stress and apoptosis via p53 and Bax activation and Bcl-2 inhibition in human astrocytoma grade IV CCF-STTG1 cells [23], and also promoted apoptosis of SH-SY5Y human neuroblastoma cells through the modulation of NF-κB and apoptotic factors [24]. However, the effect of MO leaf extract on squamous cell carcinoma (SCC) 15 cell line remains unknown.

In this study, crude EtOAc extracts and MO-derived fractions were tested for anti-SCC15 activities. Active MO-derived fractions were fraction no. 6, sub-fraction no. 6.17.2, LC-MS base peak chromatogram no. 6 identified as 3-HBI (BPC6) and 3-HBI. Our findings revealed that MO leaf extract and its active compound, 3-HBI strongly inhibited tumour cell growth and triggered apoptosis via over-expression of cleaved caspase-3 and Bax, while down-regulating the anti-apoptotic Bcl-2.

## 2. Results

### 2.1. Cellular Cytotoxicity of MO Extract, Fraction, and Sub-Fraction on Human Monocyte-Derived Macrophages and SCC15

**Figure 2:**
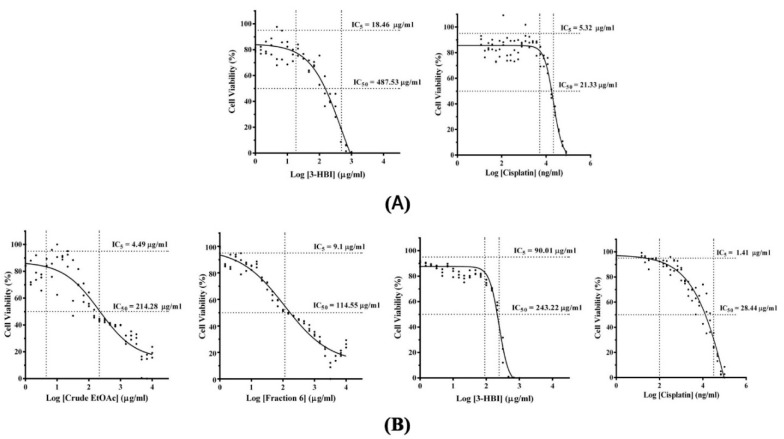
Investigation of the effect of MO crude extracts, fraction no. 6, 3-HBI and cisplatin on cell viability of MDMs and SCC15 cell lines. MTT assay was performed after cell treatment for 24 h. (**A**) IC_5_ and IC_50_ results of MDMs after treatment with different concentrations of crude extract, fraction no. 6, 3-HBI and cisplatin. (**B**) IC_50_ results for antiproliferative effect of crude extract, fraction no.6, 3-HBI and cisplatin on SCC15. IC: inhibitory concentration; 3-HBI: 3-hydroxy-β-ionone.

MTT was developed to evaluate the optimal concentration of antiproliferative effect of Moringa extracts, compound, and drugs. Human monocyte-derived macrophages (MDMs) and SCC15 were treated with different concentrations of extracts, compound, and cisplatin for 24 h. The half maximal inhibitory concentrations (IC_50_) values of Moringa extracts, compound and drug are shown in Figure 2A,B. A five percent inhibitory concentration (IC_5_) results of 3-HBI and cisplatin on MDMs were 18.46 µg/mL and 5.32 µg/mL, respectively (Figure 2A). While IC_5_ of crude ethyl acetate (EtOAc) and fraction no. 6 were 26.84 µg/mL and 84.89 µg/mL [17]. These concentrations were used as the non-toxic optimal concentration for cell culture treatment. IC_50_ values of 3-HBI and cisplatin were 487.53 µg/mL and 21.33 µg/mL, respectively (Figure 2A). The IC_50_ values of crude EtOAc, fraction no. 6, 3-HBI and cisplatin on SCC15 cell line were 214.28, 114.55, 243.22 and 28.44 µg/mL, respectively (Figure 2B). Crude EtOAc, fraction no. 6 and 3-HBI showed a strong effect in inhibiting the proliferation of SCC15 cancer cells with lower IC_50_ values compared to primary MDM.

### 2.2. Effect of MO Extract, Fraction, and Sub-Fraction on Cell Cycle of SCC15

**Figure 3:**
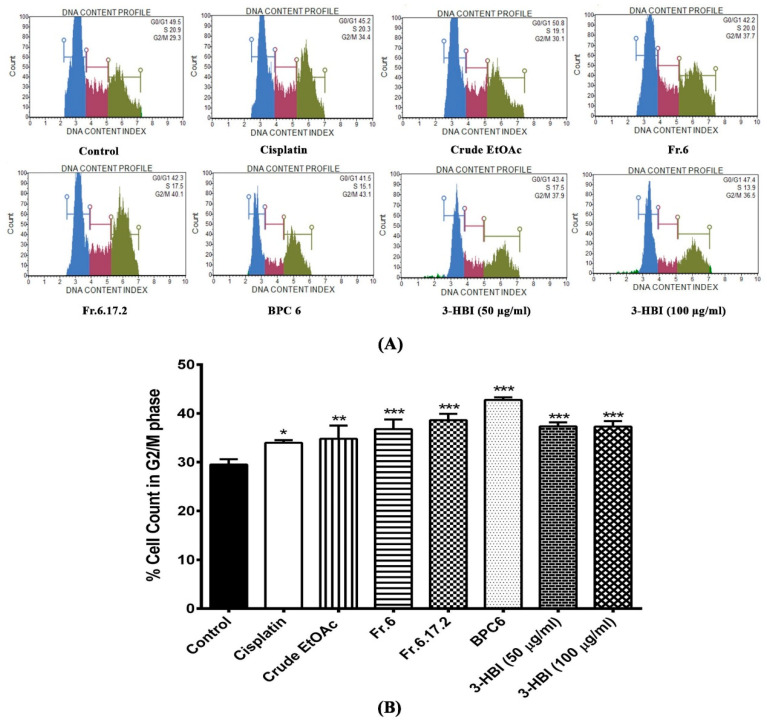
Efficacy of MO extract, fraction no. 6, sub-fraction no. 6.17.2, BPC6, and 3-HBI on distribution of SCC15 in the cell cycle analysed by Muse™ Cell Analyser. (**A**) DNA content index histogram of cell populations in each phase of the cell cycle in SCC15 cell line after treatment with cisplatin, crude EtOAc, Fr.6, Fr.6.17.2, BPC6, and 3-HBI for 24 h. (**B**) Bar graphs of percentage of cell in G2/M phase for SCC15 cell line. Data are presented as means ± SEM. * *p* ≤ 0.05, ** *p* ≤ 0.01, and *** *p* ≤ 0.001 compared to control. Control: untreated SCC15; crude EtOAc: crude ethyl acetate; Fr.6: fraction no. 6; Fr.6.17.2: sub-fraction no. 6.17.2; BPC6: base peak chromatogram no. 6; 3-HBI: 3-hydroxy-β-ionone.

Cell cycle assay was assessed using Muse™ Cell Cycle Kit. The nuclear DNA of the cell line was intercalated with propidium iodide (PI). Cells were discriminated at different phases of the cell cycle based on differential DNA content including G0/G1, S and G2/M phase. To investigate the effect of cisplatin, crude MO extract, fraction no. 6, sub-fraction no. 6.17.2, BPC6 (LC-MS base peak chromatogram no. 6 identified as 3-HBI), and 3-HBI on cell cycle progression, both untreated and treated SCC15 were investigated by Muse™ Cell Analyser. Figure 3A shows the DNA content index histogram of the control and treated cells in each cell cycle phase (G0/G1, S and G2/M). The bar graphs demonstrate that the percentage of cells in G2/M stages. After treatment with cisplatin, crude EtOAc, fraction no. 6, sub-fraction no. 6.17.2, BPC6, and 3-HBI, the G2/M enrichment phase significantly increased in SCC15 compared to the untreated control (Figure 3B). This result indicates that crude EtOAc, fraction no. 6, sub-fraction no. 6.17.2, BPC6, and 3-HBI significantly increased cell cycle arrest at G2/M phase in SCC15.

### 2.3. MO Extract, Fraction, and Sub-Fraction Induce Apoptosis in SCC15

**Figure 4:**
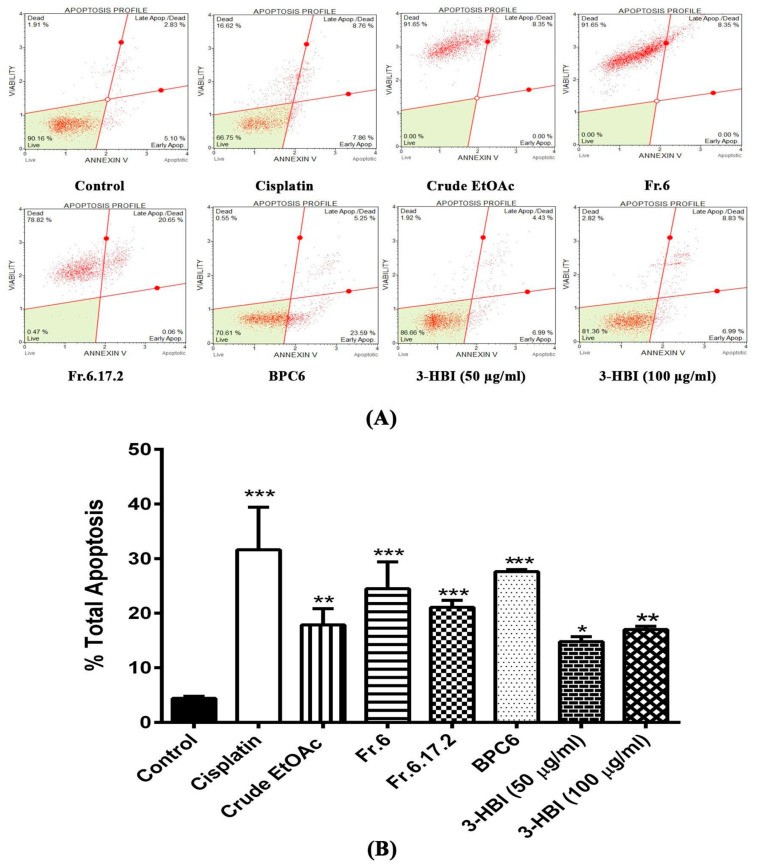
Determination of apoptosis in SCC15 cell line after treatment with MO extract, fraction no. 6, sub-fraction no. 6.17.2 BPC6, and 3-HBI for 24 h. (**A**) Dot plots of annexin V and 7-AAD dual staining showing the percentage of cell populations in each of the four quadrants. (**B**) Bar graphs showing quantitative data of percentage of total apoptotic cells. Results are presented as means ± SEM. * *p* ≤ 0.05, ** *p* ≤ 0.01, and *** *p* ≤ 0.001 compared to control. Control: untreated SCC15; crude EtOAc: crude ethyl acetate; Fr.6: fraction no. 6; Fr.6.17.2: sub-fraction no. 6.17.2; BPC6: base peak chromatogram no. 6; 3-HBI: 3-hydroxy-β-ionone.

We next evaluated the induction of apoptosis in SCC15 cell line after 24 h of treatment with MO extract, fraction no. 6, sub-fraction no. 6.17.2, BPC6, and 3-HBI. Apoptosis assay was performed by Muse™ Cell Analyser using the Muse™ Annexin V & Dead Cell Kit procedure with dot plots of SCC15 cell line stained with annexin V and 7-AAD (7-amino-actinomycin D). The first and second quadrants represent dead cells and late apoptotic cells, respectively, while the third and fourth quadrants represent live cells and early apoptotic cells, respectively, as shown in Figure 4A. These dot plots reveal that crude EtOAc, fraction no. 6 and sub-fraction no. 6.17.2 triggered late apoptosis and cell death in SCC15 cell line. Interestingly, BPC6 and 3-HBI induced early and late apoptotic cells similar to cisplatin. Figure 4B shows the statistical analysis of total apoptotic cells represented in the form of bar graphs. We observed a significantly increased percentage of total apoptotic cells (early and late apoptosis) after treatment with crude EtOAc, fraction no. 6, sub-fraction no. 6.17.2, BPC6, and 3-HBI compared to the untreated control (*p* < 0.001). The average percentage of total apoptotic cells increased from 4.41% in the control to 31.62% in cisplatin treatment. Moreover, treatment with crude EtOAc, fraction no. 6, sub-fraction no. 6.17.2, BPC6, 3-HBI (50 µg/mL), and 3-HBI (100 µg/mL) enhanced apoptotic cells to 17.85, 26.69, 21.08, 27.60, 14.76 and 17.0%, respectively. Our findings suggested that treatment with crude EtOAc, fraction no. 6, sub-fraction no. 6.17.2, BPC6, and 3-HBI strongly enhanced apoptosis in SCC15 cell line.

### 2.4. MO Extract and 3-HBI Inhibit Colony Formation of SCC15

**Figure 5:**
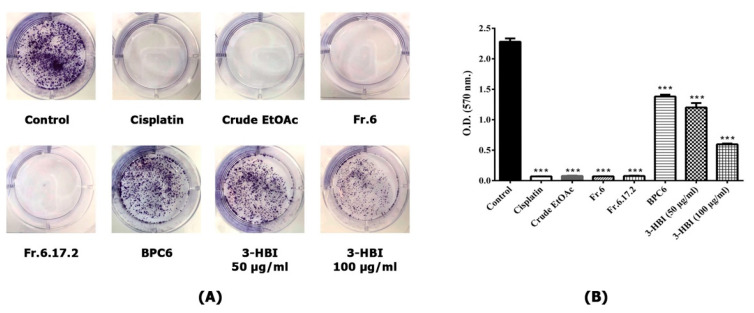
Colony formation of SCC15 cell line performed in 6-well plates with cells stained by crystal violet. (**A**) Untreated control and cells treated with MO extract, fraction no. 6, sub-fraction no. 6.17.2, BPC 6 and 3-HBI for 24 h. (**B**) Colony quantification measured by a microplate reader at OD 570 nm. Data are presented as means ± SEM. *** *p* ≤ 0.001, compared to control. Control: untreated SCC15; crude EtOAc: crude ethyl acetate; Fr.6: fraction no. 6; Fr.6.17.2: sub-fraction no. 6.17.2; BPC6: base peak chromatogram no. 6; 3-HBI: 3-hydroxy-β-ionone.

The ability of SCC15 cell line to form colonies in the presence or absence of MO extract, fraction no. 6, sub-fraction no. 6.17.2, BPC 6 and 3-HBI for 24 h was studied. SCC15 cell lines were seeded into 6-well plates and incubated for one week. Cells were fixed and stained with 0.5% crystal violet. We found that MO extract, fraction no. 6 and sub-fraction no. 6.17.2 strongly inhibited colony formation of SCC15, similar to cisplatin treatment (Figure 5A,B). Furthermore, colony formation was significantly reduced in a concentration-dependent manner by 3-HBI compared to the control, as shown in Figure 5B. A similar result was observed in SCC15 treated with BPC6. These data confirmed that MO extract, fraction no. 6, sub-fraction no. 6.17.2, BPC6 and 3-HBI showed potential to inhibit the formation of colonies in SCC15 cell lines as an important factor in cancer survival and progression.

### 2.5. MO Extract and 3-HBI Inhibit Migration of SCC15

**Figure 6:**
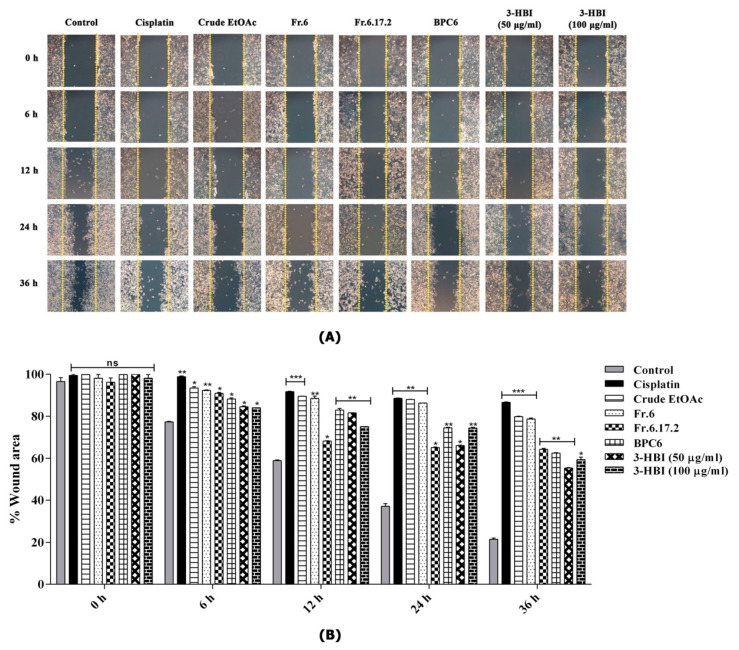
Wound closure analysis of SCC15 cell line for untreated control and cells treated with MO extract, fraction no. 6, sub-fraction no. 6.17.2, BPC 6 and 3-HBI for 36 h. (**A**) Pictures of wound area were taken at 0, 6, 12, 24 and 36 h with an inverted microscope. (**B**) Size of wound area was measured using ImageJ system software. Percentage of wound area was calculated, and bar graphs were represented as means ± SEM. ns = not significant, * *p* ≤ 0.05, ** *p* ≤ 0.01, and *** *p* ≤ 0.001 compared to control. Control: untreated SCC15; crude EtOAc: crude ethyl acetate; Fr.6: fraction no. 6; Fr.6.17.2: sub-fraction no. 6.17.2; BPC6: base peak chromatogram no. 6; 3-HBI: 3-hydroxy-β-ionone.

Wound closure assay is a method of *in vitro* study for analysing the migration of cell populations. We evaluated the migrative ability of SCC15 cell line after treatment with and without MO extract, fraction no. 6, sub-fraction no. 6.17.2, BPC6 and 3-HBI for 36 h. Cell monolayers were scratched with a similar size at baseline. We observed a significant inhibition of cell migration after treatment for 6 h. The percentage of wound area for cells treated with cisplatin, crude extract, fraction no. 6, sub-fraction no. 6.17.2, BPC6 and 3-HBI was significantly higher when compared to the control. At 36 h after wound induction, the wound area of the untreated control was almost closed. On the other hand, for the wound areas of cells treated with drugs, all extracts and compounds were still widely open (Figure 6A,B).

### 2.6. Effect of MO Extracts and Their Fractions on Apoptosis Signaling Pathway in SCC15 Cell Line

**Figure 7:**
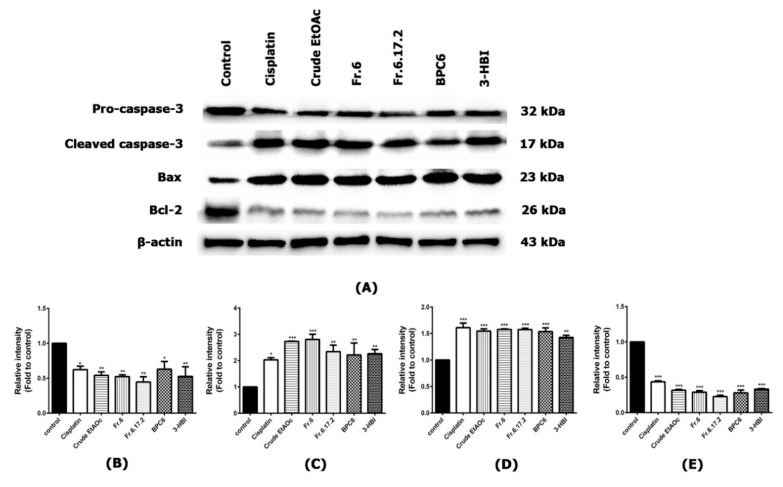
The inhibitory effect of MO extract, fraction no. 6, sub-fraction no. 6.17.2, BPC 6 and 3-HBI on the protein expressions of SCC15 cell line. Cells were treated under different conditions for 24 h. Total proteins were extracted and determined by Western blot analysis. (**A**) Band intensity of total protein levels of pro-caspase-3, cleaved caspase-3, Bax and Bcl-2. β-actin was used as a loading control. (**B**) Relative intensity of pro-caspase-3, (**C**) cleaved caspase-3, (**D**) Bax and (**E**) Bcl-2 were quantified by scanning densitometry and normalised to control. Data are shown as mean ± SEM. * *p* ≤ 0.05, ** *p* ≤ 0.01, and *** *p* ≤ 0.001 compared to control. Control: untreated SCC15; Crude EtOAc: crude ethyl acetate; Fr.6: fraction no. 6; Fr.6.17.2: sub-fraction no. 6.17.2; BPC6: base peak chromatogram no. 6; 3-HBI: 3-hydroxy-β-ionone.

Western blotting was performed to evaluate apoptosis in SCC15 cells after treatment with MO extract and its fractions. The expression of housekeeping protein β-actin was considered as an equal amount of protein was loaded. The pro-apoptotic Bax was significantly upregulated by treatment with cisplatin, MO crude extract, fraction no. 6, sub-fraction no. 6.17.2, BPC6 and 3-HBI. Moreover, they significantly decreased anti-apoptotic Bcl-2 compared to the control (*p* ≤ 0.001). Interestingly, MO extracts and their fractions reduced pro-caspase-3 expression, as well as significantly inducing the activation of cleaved caspase-3 (Figure 7A–E). Our results demonstrated that MO extracts and their fractions showed the potential to induce apoptosis of SCC15 cell line by inducing the activation of cleaved caspase-3 and Bax. Furthermore, these extracts and compound significantly decreased anti-apoptotic Bcl-2, which showed strong efficacy similar to the positive drug control.

## 3. Discussion

Cancer cells lack a regulatory system that prevents cell overgrowth. Cancer progression involves a multi-step process including self-sufficiency in proliferative signalling, uncontrolled growth, evading programmed cell death, induction of angiogenesis and inducing invasion and metastasis [6]. Several studies have examined the effects of natural compounds against tumours by inhibiting cancer proliferation and inducing apoptosis. Chikusetsu isolated from *Aralia taibaiensis* induced apoptosis in human prostate cancer [7]. Capsaicin (trans-8-methyl-N-vanillyl-6-nonenamide) is a derivative from chili peppers inhibited the migration of cholangiocarcinoma cells by downregulating metalloproteinase-9 expression [25] and promoting apoptosis by stimulated p53 and Bax expression in HCT116 human colon carcinomas [26]. Arabinogalactan and curcumin have been extensively studied for their anti-cancer properties and both natural products decreased cell growth and significantly increased Bax/Bcl2 ratio as well as cleaved-caspase3 level in MDA-MB-231 human breast cancer cells [8]. Aloe-emodin derived from *Rheum undulatum* L. inhibited proliferation and induced apoptosis via activation of caspase-9 and caspase-3 in SCC15 cells [27]. Our results showed that 3-HBI derived from MO leaf extract inhibited SCC15 cell growth and triggered apoptosis via over-expression of cleaved caspase-3 and Bax, which down-regulated the anti-apoptotic Bcl-2. Additionally, our previous finding indicated that 3-HBI of MO leaf had potent biological anti-inflammatory effects by inhibiting NF-κB translocation in LPS-treated MDMs, leading to down-regulation of pro-inflammatory mediators [17,18]. Thus, 3-HBI exhibited both anti-cancer and anti-inflammatory activities and might be a novel effective therapeutic drug for head and neck cancer since NF-κB signalling pathways are targeted for therapeutic applications in many cancers including HNSCC. Accordingly, the major class of cellular targets controlling NF-κB consists of chemokines, regulators of apoptosis, cell proliferation and cell cycle regulators [28,29,30]. Curcumin has also been shown to inhibit NF-κB. This regulates several cellular processes including cell growth and survival by suppressing Bcl-2 and cyclin D1, IL-6, COX-2, and MMP-9 protein expression in HNSCC [31]. Erlotinib and EGCG of green tea extract synergistically inhibited HNSCC growth via inhibiting NF-κB in a p53-dependent manner [32].

MO leaf has various medicinal properties including antioxidant, anti-inflammatory, antiulcer, hepatoprotective activities, antibacterial and antifungal activities [33]. Cancer research involving Moringa leaf has been conducted in both *in vitro* and *in vivo* such as Moringa in MDA-MB-231 breast cancer cells, human HCT8 colon cancer cells and mouse melanoma [19,21,34]. This study evaluated the anti-cancer properties of Moringa leaf extract and 3-HBI bioactive compound including cell viability, cell cycle, apoptosis, migration, and colony formation of SCC15 cell line. The MTT assay was performed to evaluate cell viability of human MDMs and SCC15 cell line in the presence of MO leaf extract and its fractions. Our results showed that concentrations of the extract and compound caused cytotoxicity of SCC15 but non-toxicity in normal cells. Interestingly, this result concurred with our findings, indicating cell cycle arrest and an increase in cell apoptosis. We found that MO extracts and fractions caused a significant increase in cell population at the G2/M phase compared to the untreated control (*p* ≤ 0.001). A previous study by Al-Asmari et al. (2015), showed similar findings with cell cycle arrest at the G2/M phase in MDA-MB-231 and HCT-8 cancer cell lines after treatment with Moringa leaf extract [21]. Cell migration and colony formation are hallmarks of tumour progression. Wound closure assays allow the observation of cell migration in confluent monolayer cell cultures, while colony formation assay is an *in vitro* technique for studying the survival and proliferation of cancer cells based on the ability of single cells to grow into colonies [35]. In this study, MO leaf fractions were able to significantly inhibit colony formation and cell migration of SCC15 cell line.

Apoptosis is programmed cell death that generally occurs in tissue during development as a homeostatic mechanism. Apoptosis is activated via two pathways, commonly known as intrinsic and extrinsic. The intrinsic pathway is initiated by pro-apoptotic proteins such as Bax and Bad that damage the mitochondrial membrane, leading to release of cytochrome C. Then, the formation of apoptosome complex activates procaspase-9 and stimulates caspase 3-6-7, resulting in apoptosis. The extrinsic pathway is initiated by death receptors at the cell surface to the intracellular signalling pathways. Then, caspase 8 is activated leading to stimulate downstream caspase 3-6-7 [36,37]. In various tumours, pro-apoptotic members are normally downregulated while anti-apoptotic factors are upregulated [6]. Our study showed that cell apoptosis in SCC15 cell line was strongly induced by MO leaf extracts and their fractions. Our results were further confirmed through activating the apoptosis signalling pathway by significantly increasing pro-apoptotic (BAX) and cleaved caspase-3 while suppressing the expression of anti-apoptotic protein Bcl-2 compared to the untreated control. These results indicated that 3-HBI bioactive compound of MO leaf showed strong anti-cancer activity by inducing apoptosis in SCC15 cell line via a caspase-dependent mechanism.

## 4. Materials and Methods

### 4.1. Preparation of Moringa Oleifera Leaf Extracts and Compound Identification

Moringa leaves were ground to a powder and extracted at room temperature with EtOAc as described in our previous study [17]. Then, 128 g of crude EtOAc extract was obtained after evaporation of the solvent. Fractions and sub-fractions were separated from the crude extract using flash column chromatography (Merck, Darmstadt, Germany). Gradient elution was performed by solvent system with increasing the polarity gradually including hexane, hexane-EtOAc and EtOAc-Methanol (MeOH). Apocarotenoid monoterpene namely 3-hydroxy-β-ionone, an active compound was identified from the Moringa sub-fraction by LC-ESI-QTOF-MS/MS (Agilent Technologies, Inc., Singapore). The crude EtOAc extracts and MO-derived fractions (fraction no.6, sub-fraction no. 6.17.2, BPC6 and 3-HBI) were tested for anti-cancer activities.

### 4.2. Monocyte Isolation

Human MDMs were used as a primary normal cell control. Buffy coat was obtained from the Blood Bank, Naresuan University Hospital, Phitsanulok, Thailand. Ethics approval was obtained from the Human Ethics Committee of Naresuan University (IRB no. 1013/60). Buffy coat was diluted with Hank’s balanced salt solution (HBSS) and then overlaid on 5 mL Lymphoprep (Stemcell Technologies, Singapore) and centrifuged at 2000 rpm for 30 min. The mononuclear cell layer was collected and washed twice with HBSS buffer. Peripheral blood mononuclear cells (PBMCs) were collected and suspended in 5 mL of RPMI medium. Then, the monocytes were separated by size sedimentation centrifugation using Percoll (GE Healthcare Bio-Sciences AB, Uppsala, Sweden). The PBMC suspension was carefully overlaid on 10 mL of 46% Percoll solution and centrifuged for 30 min at 2000 rpm. Monocytes between Percoll were collected and washed with HBSS, followed by centrifugation for 10 min at 1300 rpm. Isolated monocytes were cultured in RPMI 1640 and supplemented with 10% fetal bovine serum (FBS) and 1% Antibiotic-Antimycotic purchased from Gibco^TM^ (Thermo Fisher Scientific, Inc., New York, NY, USA). Cells were incubated at 37 °C with 5% CO_2_ for 2 weeks with media replacement every 3 days.

### 4.3. Cell Line and Culture Conditions

Squamous cell carcinoma 15 (ATCC^®^ CRL-1623™) was purchased from ATCC. Cells were cultured in Dulbecco’s modified Eagle’s medium (DMEM) and Ham’s F12 medium (Caisson Labs Inc., Smithfield, UT, USA) containing 10% FBS. Cells were incubated at 37 °C with 5% CO_2_ with medium renewal every 2–3 days. SCC15 cells were seeded in 24-well plates at a density of 5 × 10^4^ cells/well and incubated for 24 h. The cells were treated with MO crude extracts, their fractions and 3-HBI (Santa Cruz Biotechnology, Inc., Dallas, TX, USA) for 24 h. Untreated and Cisplatin (Sigma-Aldrich, St. Louis, MO, USA) treated SCC15 cells were used as control conditions.

### 4.4. Cell Viability Assay

MTT assay was used to determine the growth inhibitory role of MO leaf extract. MDMs and SCC15 were seeded in a 96-well plate at a density of 1 × 10^4^ cells/well and treated with various concentrations of extract, compound, and cisplatin. Cells were incubated at 37 °C for 24 h. Then, 50 µL of MTT (0.5 mg/mL) (Invitrogen, Carlsbad, CA, USA) in medium-free serum was added in all samples and they were incubated at 37 °C for 3 h. MTT reagent was removed and formazan crystals were dissolved in 100 µL of DMSO. The absorbance of formazan solution was measured at 590 nm by a microplate reader (PerkinElmer, Inc., Waltham, MA, USA). This method followed previous protocol [38]. IC_50_ of cisplatin, crude EtOAc extract and 3-HBI were calculated by concentration response relationships/sigmoidal curve fitting analysis. IC_5_ was selected as non-toxic for cellular experiments.

### 4.5. Cell Cycle Analysis

To confirm the growth inhibitory role of MO extracts and derivative compounds, cell cycle assay was analysed using Muse™ Cell Cycle Kit (Merck, Darmstadt, Germany) following the manufacturer’s protocol. Experimental conditions of SCC15 were harvested using trypsin/EDTA solution (Thermo Fisher Scientific) and incubated at 37 °C for 5 min. Then, 200 µL of completed DMEM HamF12 were added to stop the reaction of trypsin. Cells were aspirated and centrifuged at 1500 rpm for 5 min, then fixed with 70% ethanol and incubated for at least 3 h at −20 °C. Cells were washed twice with cold PBS (phosphate buffer saline), resuspended in 200 µL of Muse™ Cell cycle reagent, mixed gently and incubated for 30 min at room temperature in the dark. Cell cycle stage was then analysed by Muse™ Cell Analyser.

### 4.6. Cell Apoptosis Analysis

Muse™ Annexin V & Dead Cell Kit (Merck, Darmstadt, Germany) was used for the apoptosis study. SCC15 cells from all experimental conditions were harvested by trypsin/EDTA solution as described in cell cycle assay. Cells were washed in PBS and resuspended in medium with 1% FBS. Then, 100 µL of Muse™ Annexin V & Dead Cell Reagent were added, mixed gently, and incubated for 20 min at room temperature in the dark. Cell apoptosis was measured using Muse™ Cell Analyser following the manufacturer’s protocol.

### 4.7. Colony Formation Assay

Colony formation assay was used to study the potentiality of a single cell forming colonies. This assay followed the previous description [35]. SCC15 cell line was seeded into 6-well plates at a density of 500 cells/well and incubated for 24 h in standard culture conditions at 37 °C. Cells were then treated with drugs and Moringa extracts including crude EtOAc, fraction no. 6, sub-fraction no. 6.17.2, BPC6 and 3-HBI for 24 h. Cells were incubated for 1 week with media replacement every 3 days and then fixed with 10% neutral buffer formalin solution for 30 min. The fixative reagent was removed, and the cells were stained with 2 mL of 0.5% crystal violet and incubated for 60 min at room temperature on a rotator. Cells were washed 4 times in a stream of tap water and the plate was air dried for at least 2 h at room temperature. Then, 2 mL of methanol were added to each well and the plate was incubated for 20 min at room temperature on a rotator. Optical density of each well was measured at 570 nm with a microplate reader (PerkinElmer, Inc.).

### 4.8. Wound Closure Assay

Cell migration of SCC15 cell line was evaluated by wound closure assay modified from a previous method [39]. SCC15 cells were seeded into 6-well plates at a density of 1 × 10^6^ cells/mL and incubated at 37 °C until reaching 80% confluence as a monolayer. The cell monolayer was scraped in a straight line with a SPLScar Scratcher (SPL Life Sciences, Gyeonggi-do, Korea). Detached cells were removed and washed twice with 1 mL of medium. Cells were treated as described in the colony formation assay and incubated for 36 h. Snapshots were taken of the experimental cell plates at several time points including 6, 12, 24 and 36 h using an inverted microscope (Carl Zeiss Microscopy GmbH, Jena, Germany). The distance of the wound area was analysed by ImageJ system software.

### 4.9. SDS-PAGE and WESTERN BLOT ANALYSIS

Total proteins of SCC15 cell line from each condition were extracted by ice-cold RIPA lysis buffer (Bio Basic Inc., New York, NY, USA) in the presence of Halt Protease/Phosphatase Inhibitor Cocktails (Thermo Fisher Scientific) and centrifuged at 12,000 rpm for 15 min at 4 °C. Quantification of total protein concentration was performed by Bradford Coomassie-binding, colourimetric method. Protein extract was mixed with an equal volume of 4X Laemmli loading buffer and heated to denature at 95 °C for 5 min. Samples were loaded into wells of 12% SDS-polyacrylamide gel electrophoresis (PAGE) with proteins separated according to molecular weight and transferred to a polyvinylidene fluoride membrane (Bio-Rad Laboratories, Inc., Hercules, CA, USA). For Western blot analysis, the membrane was blocked for 2 h at room temperature with blocking buffer containing 5% bovine serum albumin (Capricorn Scientific GmbH, Ebsdorfergrund, Germany) in Tris-buffered saline with Tween 20 (TBST) buffer. The membrane was blotted using primary antibodies specific to cleaved-caspase 3 (Asp175, p17) (Affinity Biosciences, Cincinnati, OH, USA), β-actin, pro-caspase 3, Bax and Bcl-2 (Santa Cruz Biotechnology, Inc., Dallas, TX, USA) overnight at 4 °C on a rotator. The membrane was washed with TBST and incubated with horseradish peroxidase-conjugated goat anti-mouse IgG (H + L) secondary antibody (Thermo Fisher Scientific) for one hour at room temperature. The membrane was observed by soaking in chemiluminescence substrate for 5 min and placed in a ChemiDoc XRS+ Imaging System (Bio-Rad Laboratories, Inc., Hercules, CA, USA). The chemiluminescence signal of the blotted membrane was detected by Image Studio Lite software (LI-COR Corporate, Lincoln, NE, USA).

### 4.10. Statistical Analysis

All experiments were performed in three independent batches of experiments to provide accurate results. One-way ANOVA and the Bonferroni multiple comparisons test were used for data analysis with GraphPad Prism software. A confidence interval of 95% (*p* = 0.05) was used in all statistical analyses.

## 5. Conclusions

**Figure 8:**
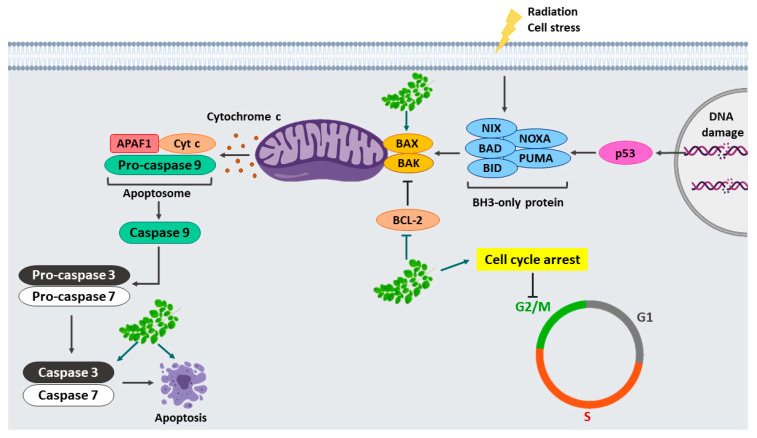
Apoptosis signalling pathway and cell cycle arrest in response to Moringa extract and 3-HBI. MO crude extract and 3-HBI induced G2/M cell cycle arrest and apoptosis in SCC15 cell line by increasing the Bax and cleaved caspase-3 expression and downregulating the levels of Bcl-2.

Moringa extract and its compound, 3-HBI suppressed cell proliferation and induced apoptosis in SCC15 cell line through the activation of cleaved caspase-3 and Bax as well as suppressing anti-apoptotic factor, Bcl-2. Moreover, treatment with MO extract and 3-HBI significantly increased the G2/M phase arrest of cell cycle progression in SCC15 (Figure 8). We observed a significant inhibition of cell migration as well as colony formation in SCC15 cells after treatment with crude extract and 3-HBI. Our findings suggest that MO extract and 3-HBI have potential as an anti-cancer treatment. This is the first report concerning MO extract and 3-HBI activity against SCC15 cell line.
